# A Survey of Deep Convolutional Neural Networks Applied for Prediction of Plant Leaf Diseases

**DOI:** 10.3390/s21144749

**Published:** 2021-07-12

**Authors:** Vijaypal Singh Dhaka, Sangeeta Vaibhav Meena, Geeta Rani, Deepak Sinwar, Kavita, Muhammad Fazal Ijaz, Marcin Woźniak

**Affiliations:** 1 Department of Computer and Communication Engineering, Manipal University Jaipur, Dehmi Kalan, Jaipur, Rajasthan 303007, India; vijaypalsingh.dhaka@jaipur.manipal.edu (V.S.D.); sangeeta.yad@gmail.com (S.V.M.); geeta.rani@jaipur.manipal.edu (G.R.); 2 Department of Computer Science and Engineering, Chandigarh University, Mohali, Punjab 140413, India; kavita@ieee.org; 3 Department of Intelligent Mechatronics Engineering, Sejong University, Seoul 05006, Korea; 4 Faculty of Applied Mathematics, Silesian University of Technology, 44-100 Gliwice, Poland; marcin.wozniak@polsl.pl

**Keywords:** convolutional neural networks, deep learning, agriculture, leaf, disease, survey

## Abstract

In the modern era, deep learning techniques have emerged as powerful tools in image recognition. Convolutional Neural Networks, one of the deep learning tools, have attained an impressive outcome in this area. Applications such as identifying objects, faces, bones, handwritten digits, and traffic signs signify the importance of Convolutional Neural Networks in the real world. The effectiveness of Convolutional Neural Networks in image recognition motivates the researchers to extend its applications in the field of agriculture for recognition of plant species, yield management, weed detection, soil, and water management, fruit counting, diseases, and pest detection, evaluating the nutrient status of plants, and much more. The availability of voluminous research works in applying deep learning models in agriculture leads to difficulty in selecting a suitable model according to the type of dataset and experimental environment. In this manuscript, the authors present a survey of the existing literature in applying deep Convolutional Neural Networks to predict plant diseases from leaf images. This manuscript presents an exemplary comparison of the pre-processing techniques, Convolutional Neural Network models, frameworks, and optimization techniques applied to detect and classify plant diseases using leaf images as a data set. This manuscript also presents a survey of the datasets and performance metrics used to evaluate the efficacy of models. The manuscript highlights the advantages and disadvantages of different techniques and models proposed in the existing literature. This survey will ease the task of researchers working in the field of applying deep learning techniques for the identification and classification of plant leaf diseases.

## 1. Introduction

There is an exponential increase in population around the globe. As per the report published in [1], the population is expected to reach 8.5 billion by 2030. Thus, there is a solid requirement to maximize the production of the agriculture industry for fulfilling the needs of the increasing population. The growth of bacteria, viruses, fungi, nematodes, and other microorganisms is increasing due to weather conditions such as temperature, humidity, and precipitation. Plants become more prone to diseases due to a large number of pathogens in their surroundings. Attacks of pests and diseases are significant causes of the reduction in crop production. Precise prediction of plant diseases well in time helps to apply suitable prevention and protection measures. Hence, it helps improve the yield quality and increase crop productivity.

Diseases in plants are detected by various symptoms such as lesions, changes in color, damaged leaf, damage in the stem, abnormal growth of stem, leaf, bud, flower and/or root, etc. In addition, leaves show symptoms such as spots, dryness, pre-mature falls, etc., as an indicator of disease [2]. Analyzing these observable symptoms is an effective way to detect plant diseases. The traditional disease detection approach is a visual examination of a plant by a trained or experienced person(s). However, the approach requires sound knowledge and expertise in the field of disease detection. Moreover, it can result in erroneous predictions due to visual illusions and biased decisions [3]. Thus, the approach is not a practical solution for large agricultural land.

The traditional approach’s limitations have motivated the researchers to propose technological solutions for disease prediction in plants. Artificial Neural Network (ANN) [4], Support Vector Machine (SVM) [5], Bayes classifier [6], Random Forests [7], K-Nearest Neighbor (KNN) [8], etc. are used for developing automated disease detection models. A disease detection model completes its task in five steps: plant leaf image acquisition, pre-processing of images, segmentation, feature extraction, and classification of different diseases. Existing models are effective in disease identification. However, disease classification accuracy depends on human expertise in leaf image acquisition [9]. Thus, there is a scope of improvement in these models according to the type of available datasets and experimental conditions. Therefore, the researchers in [10] focus on enhancing the efficiency and accuracy of the above-said models.

Due to the availability of a plethora of research works in applying machine learning and deep learning models for predicting plant diseases, it becomes difficult for researchers to select an effective model according to the dataset, parameters, hardware configuration, and experimental conditions. Thus, there is a demand for a comprehensive survey of the existing literature that can assist the researchers in identifying a suitable model for data pre-processing, prediction, and classification of plant diseases. Therefore, the authors present an extensive survey of the pre-processing techniques, Deep Convolutional Neural Network (DCNN) architectures, DCNN frameworks, and Optimization techniques in this manuscript. In addition, the manuscript highlights the advantages, drawbacks, and applications of the deep learning models developed in the field of identification and classification of plant leaf diseases.

The remaining sections of this paper are organized as follows: Section 2 describes the literature survey. It includes the technologies applied for automatic disease prediction. This section also includes the problem definition, challenges, and progress made during the last 5 years in the field of plant leaf disease identification. Section 3 presents the comparative analysis of various pre-processing techniques, commonly used CNN models for plant disease detection, various optimization techniques, and different frameworks used for plant disease detection and classification. Finally, Section 4 presents the discussion of the works, issues, and challenges of current approaches, conclusions drawn, and directions for future work.

## 2. Materials and Methods

**Figure 1:**
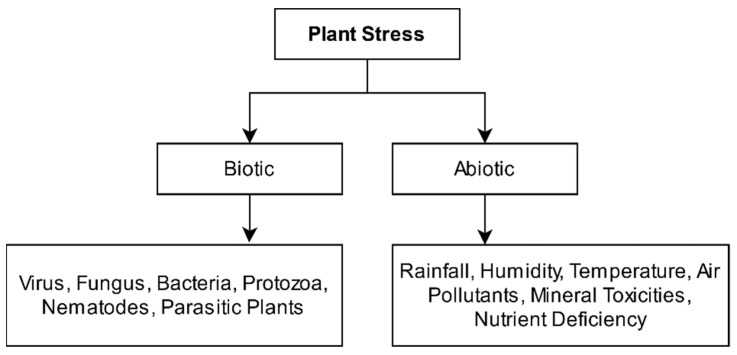
Types of plant stress.

The literature study shows that nutrition deficiency, attack of microbes, rodents, unfavorable environmental conditions, etc., are the leading causes of plant diseases [11]. These factors lead to plant stress, impairment in the structure or functioning of a plant. Plant stress is broadly categorized into two categories: biotic and abiotic, as shown in Figure 1. Biotic stress originates from living organisms such as fungi, bacteria, protozoa, viruses, nematodes, or parasitic plants. The agents causing biotic stress restricts plants to nutrients that lead to severe damage to the plants. In response to the attack, plants counterattack to recover with the help of various strategies, i.e., plant’s genetic code. On the other hand, abiotic stress arises from non-living influences, i.e., unfavorable atmosphere, lack of soil nutrients, extreme sunlight, variation in temperatures, excessive or low rainfall, inappropriate oxygen, moisture levels, deficiency, or excess essential mineral(s). Biotic stress is infectious, transmissible, and more dangerous than abiotic stress [12]. Sometimes, due to the heavy chewing of plant leaves by insects, the total area of a leaf is reduced to a great extent, which results in a reduction of photosynthesis as well. Azimi et al. [13] presented a deep learning-based approach for plant leaf stress identification caused by nitrogen deficiency. On the other hand, Noon et al. [14] presented a plant leaf stress identification survey using deep learning techniques.

A diseased plant shows symptoms such as a change in color, shape, size, retardation in growth, etc. These symptoms vary at different stages of a disease. At the transition stage, disease-causing factor(s) start affecting a healthy plant, and there is a gradual appearance of symptoms for a disease. At this stage, it is difficult to distinguish a healthy plant from a diseased plant. Additionally, there are fair chances that a disease weakens the immune system and multiple diseases attack the plant. Thus, there is an appearance of similar symptoms for two or more diseases [15].

Moreover, environmental factors such as temperature, wind, humidity, exposure to sunlight, and other meteorological phenomena can alter the symptoms of a disease. These factors lead to the variations in shape, color, and size of the disease-affected region(s). In such cases, it becomes challenging to identify a disease by merely examining a plant or plant part with naked eyes [16]. On the other hand, the advanced techniques of Artificial Intelligence (AI), such as Convolutional Neural Networks (CNN) and Deep Convolutional Neural Networks (DCNNs), can minimize human intervention and give good accuracy in disease identification. Nevertheless, irrelevant information such as background with dead leaves, soil, branches, leaves of other plants, weeds, insects, etc., in an image is a challenge in identifying disease.

Moreover, the quality of a plant image is highly dependent on the illumination conditions, overcast conditions, the position of the sun and angle of reflection, etc., while capturing an image. A poor-quality image with low contrast does not give sufficient information for disease identification. More challenges in applying AI and DCNNs are building a suitable model according to the dataset, collecting a vast dataset for training a model, finding the optimal number of layers in a model, and determining the number of neurons in each layer. In addition, determining the optimal number of parameters to be fed to CNN is a challenging task [17].

Deep learning models can face the problem of vanishing gradients during the training phase. During the adjustment of parameters, the gradient of the loss function may approach zero, which makes the network difficult to train. The initial layers play a vital role in recognizing the basic features of the input data. A small gradient means that the parameters of the initial layers will not be updated effectively, leading to the overall inaccuracy in the entire network. Thus, the accuracy of a model depreciates by increasing the depth of a network beyond a threshold value. Wang et al. claimed that the accuracy of a shallow network decreases by increasing the depth of a network beyond eight [18]. At the time of training of Deep Neural Networks, the output of one layer is fed as an input to the next layer. A change in a layer’s parameters leads to variation in the distribution of input data to the subsequent layers. Thus, it causes the Internal Covariate Shift problem. The problem slows down the training process. It requires the vigilant initialization of parameters and lower learning rates. To minimize the Internal Covariate Shift problem, the authors applied the Batch Normalization technique [19]. Batch Normalization permits the usage of much higher learning rates and less concern about initialization. It tries to normalize the inputs fed to each hidden layer. Therefore, the distribution of inputs is relatively constant. This improves the overall accuracy and rate of learning in deep networks [20].

### Recent Developments in Plant Leaf Disease Identification and Classification

A survey of the existing literature shows extensive use of image processing techniques and machine learning algorithms for the detection and classification of plant leaf diseases in the last 2 decades. Table 6 shows a comparative analysis of deep CNN models applied for the identification of plant leaf diseases. From 2015 onwards, deep learning models gained popularity due to their high accuracy in classifying plant leaf diseases. The comparison of machine learning and deep learning models is shown in Table 1.

## 3. Comparative Analysis

This section presents a detailed comparison of the techniques applied for pre-processing, different convolutional neural networks applied on different datasets, architectures, optimization techniques, and frameworks applied for automating the disease prediction.

### 3.1. Pre-Processing Techniques

Captured images of plant leaves contain noise, undesirable background, low illumination, etc. Applying classification techniques on these captured images does not give accurate results [2]. Therefore, there is a need to apply pre-processing techniques on raw datasets before feeding a dataset as an input to a CNN model. This is important to accelerate the training process and improve classification accuracy.

**Figure 2:**
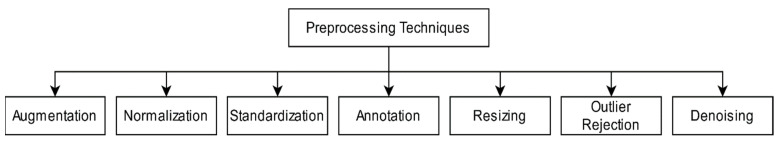
Categories of pre-processing techniques.

Pre-processing includes resizing images, converting colored images into grayscale images, normalization, augmentation, cropping, and extracting Region of Interest (ROI), etc. Figure 2 displays the categories of the most used pre-processing techniques.

The study of literature evidences the availability of a plethora of pre-processing techniques under each category. Applying pre-processing techniques transforms the dataset so that a CNN model precisely and efficiently classifies a given dataset. Exemplified as conversion of Red–Green–Blue (RGB) images into grayscale images makes the training process more manageable and faster as a single color channel has lower computational requirements than multiple color channels [21,22,23]. Applying Principal Component Analysis (PCA) transforms data into a compressed space with a smaller number of dimensions. Zero-Phase Component Analysis (ZCA), a whitening method, is similar to PCA. It is applied to highlight the features and structures for making the learning process easier [17]. Cropping on images is applied to highlight the ROI [24,25,26]. Contrast stretching is applied before segmenting a region using the correlation coefficient method [27]. This strengthens the visual quality of a diseased region. Otsu’s algorithm is applied to perform the segmentation of leaf images [28]. The following discussion reveals the details about existing pre-processing techniques.

#### 3.1.1. Resizing

Images gathered from different sources differ in size. The difference in size adds to the training time of the model. Therefore, there is a requirement to resize the captured and/or collected images before applying a CNN model. The padding of zeros in small images and cropping of large images are valuable techniques for maintaining uniformity in the size of images. Resizing of images is completed according to an input layer of the CNN model, exemplified as an image resized to 299 × 299 in the Inception-v3 model [29] and Xception model [30]. In the Visual Geometry Group (VGGNet), as discussed in [31], ResNet presented in [32], and DenseNet architecture discussed in [33], images are resized to 224 × 224. In AlexNet, images are resized to 227 × 227 [34]. Researchers in [23,24,25,28,35] applied automatic resizing on images by writing scripts in programming languages such as Python and Java. The written script automatically reduces the dimensions of fed images to 256 × 256. Authors in [17] reduced the dimensions of RGB images from 5760 × 3840 to 512 × 512 during retainment of the model with features of input images. Authors in [22,36] applied the resizing of images to reduce the size from 2048 × 1536 and 2736 × 1824 pixels to 224 × 224 pixels. Authors in [21] used plant leaf images of 60 × 60 pixels. Authors in [26] used images of 200 × 200 pixels, [37] 800 × 600 pixels, and [38] used images of 832 × 832 pixels to enhance the efficiency of image processing techniques and to minimize the computational cost. The above discussion shows that resizing images is essential to reduce the time of training a model and perform classification.

#### 3.1.2. Augmentation

The performance of Deep Neural Networks is highly dependent on the amount of data used for training a model. In case a small dataset is available, then there is a need for data augmentation. There are multiple techniques of data augmentation such as brightness change, horizontal and vertical shift, cropping, PCA jittering [39], shearing, flipping, and zooming of training images [18], rotation [22,40], and affine transformations [25]. In addition to the above-stated traditional techniques, advanced techniques such as Generative Adversarial Networks (GANs) and Neural Style Transfer (NST) are applied to augment deep learning. These augmentation techniques increase a training data set by artificially generating modified forms of images [41]. Thus, they prevents overfitting in a network and enhances the performance of a model [42,43].

#### 3.1.3. Normalization and Standardization

Normalization is a process of scaling dimensions or values of pixel intensity in a particular range. Exemplified as an 8-bit RGB image, pixel values are integer numbers ranging from 0 to 255. The learning process of a CNN is disturbed when large input values are multiplied by small values of weights. Therefore, there is a requirement to normalize the values of pixels. This is completed by dividing all pixel values by the largest value, i.e., 255. It gives all values in a range from 0 to 1 across all channels without disturbing the view of images [20]. Normalization helps in eliminating distortions caused by lights and shadows in an image. It gives equal importance to each feature and improves the model’s learning rate, quality, and accuracy. There are different ways of performing normalization, such as Decimal scaling, Min–Max normalization, and Z-score normalization.

Min–Max normalization scales data into a range from 0 to 1 as given in Equation (1). Here, the values of new_min_ and new_max_ are 0 and 1 respectively, x is the value of an attribute, max is the maximum value of the given attribute, and min is the minimum value of the given attribute. This technique gives stable gradients. However, it lacks handling outliers. Min–Max normalization scales data into a range from 0 to 1 as given in Equation (1). Here, the values of new_min_ and new_max_ are 0 and 1 respectively, x is the value of an attribute, max is the maximum value of the given attribute, and min is the minimum value of the given attribute. This technique gives stable gradients. Nevertheless, it lacks handling outliers.

(1) $${\mathrm{new}}_{x}=\frac{x-\min}{\max-\min}\left({\mathrm{new}}_{\max}-{\mathrm{new}}_{\min}\right)+{\mathrm{new}}_{\min}$$

Z-score normalization or standardization involves the rescaling of pixel values. It performs zero centering of data by subtracting the mean value from each pixel and then dividing each dimension by its standard deviation, as given in Equation (2). Here, z is the standardized value, x is the value of an attribute, the mean, and the standard deviation.

(2) $$z=\frac{\left(x-\mu \right)}{\sigma }$$

#### 3.1.4. Annotation

Annotation is a technique of assigning labels to images for training a model. For detecting diseases in plant leaves, experts with sound knowledge about the diseases perform the annotation [43]. There are different techniques of annotation, such as bounding box annotations and pixel-wise image annotations. The bounding box annotation is a commonly used annotation approach. In this approach, a tight rectangle or cuboid is fitted around the targeted object. The drawback of this technique is that it includes additional noise in the bounded box. This technique also faces difficulty in the case of occluded objects. In pixel-wise image annotation, point-by-point object selection is made. Pixel-wise image annotation takes more time than bounding box annotations [44] because it makes point-by-point object selection through the edges of objects. This technique is costly, time-consuming, and prone to human errors.

#### 3.1.5. Outlier Rejection

Outlier rejection involves ignoring invalid or irrelevant images from a dataset. Exemplified as low resolution, low values of intensity, blurriness, noise, irrelevance, duplicate images are criteria for rejection [25]. Authors in [45] developed a CNN model, namely, organNet, for the removal of unwanted or invalid images from a given dataset.

#### 3.1.6. Denoising

Denoising is the removal of noise from an image without adversely affecting the features of an image. It improves the performance of an image classification technique in a noisy environment. Researchers apply different denoising techniques such as Gaussian filter, Mean filter, Wiener filter, Median filter, Small-window median filter, and Bilateral smoothing filter, etc.

Gaussian filter, a popular denoising algorithm, is used to blur an image and removes noise and details related to the noise by applying the Gaussian function. A median filter is a non-linear filter. It removes the non-additive type of noise. In the Median filter, a 7 × 7, 5 × 5, or 3 × 3 filter of pixels is scanned over the pixel matrix of an image. The median of all pixel values replaces the central pixel in the window. It is effective in preserving sharp edges. It is useful in image processing for reducing salt and pepper noise. Salt-and-pepper noise represents arbitrarily occurring white and black pixels. An image containing salt-and-pepper noise has dark pixels in the bright regions and bright pixels in the dark regions. Mean filter and Wiener filter gives the best performance with Gaussian noise filtering. Cruz et al. applied a small-window median filter for removing noise in the leaf image dataset [28].

The above discussion indicates that pre-processing techniques such as resizing, augmentation, normalization and standardization, annotation, outlier rejection, and de-noising play an essential role in transforming raw datasets into a desired form. The transformed dataset is fed to a CNN model as input for yielding accurate classification results faster. The pre-processing techniques differ in mechanism of working and types of dataset. Each technique has its advantages and drawbacks. The comparison of pre-processing techniques, as shown in Table 2, gives an idea to researchers to select an appropriate pre-processing technique.

### 3.2. Convolutional Neural Networks

**Figure 3:**
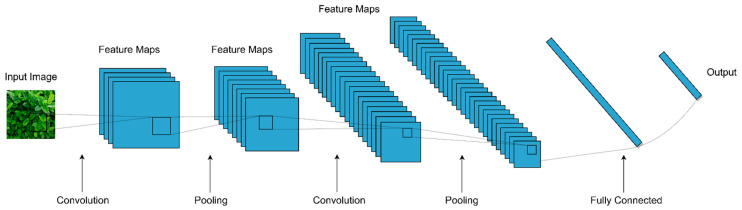
Structure of a typical CNN model.

Convolutional Neural Networks were discovered in 1962 by Hubel and Wiesel [46]. The survey of the existing literature reveals that CNNs are the most popular deep learning models used for the classification of image data [47]. The structure of a CNN is inspired by the structure of the receptive field of the visual cortex in humans. A CNN is a feed-forward Neural Network. A hierarchical network is composed of multiple layers, namely, convolution, pooling, and fully connected layers. The structure of a CNN model is shown in Figure 3.

CNN architectures are trained through back-propagation algorithms for tuning the weights and biases of a network. As a result, it reduces the value of the cost function. In the late 1980s, the CNN models were applied to recognize the handwritten zip code digits taken from the U.S. Postal Service [48]. On extending applications of CNN for detecting diseases of plant leaves, it is observed that CNN scans a dataset containing leaf images and identifies and extracts the essential features before classifying the diseases. The Decision-making technique of CNN is similar to human beings. It captures the textures and color lesions of plant leaves for disease identification [49]. The feature learning power of CNN automatically detects the essential features directly from a leaf image through many non-linear filters. Thus, the overall performance of a model becomes better than models using hand-engineered features [50].

### 3.3. Datasets and CNN Models

Researchers used various datasets for the detection and classification of plant diseases by applying deep learning models. Mohanty et al. trained a CNN model using the dataset ‘PlantVillage’ developed by the authors [51]. For preparing this dataset, the authors plucked leaves from plants and kept the plucked leaves on a black or grey background sheet. They captured images of leaves using a digital camera (Sony DSC—Rx100/13 20.2 megapixels) under different environmental conditions, such as strong sunlight, cloud, and shade. In addition, they set ideal conditions of illumination, background, distance, and angle of capturing an image. The authors used a wide variety of 54,306 images of 14 crops with 26 different diseases to train the CNN model. Mohanty et.al performed a set of experiments using three different sets of the PlantVillage dataset. The first set refers to the original version of the ‘PlantVillage’ dataset containing colored images. The second set refers to the gray-scaled version of the ‘PlantVillage’ dataset. The third set includes images of segmented leaves where extra background information is removed from the PlantVillage dataset. They trained the CNN model with 80% data and tested the model for 20% data of the total dataset. They achieved the highest accuracy of 99.35% on colored leaf images by using GoogLeNet architecture with transfer learning [23]. The details about experiments on three sets of PlantVillage datasets are discussed in [51].

Similarly, L. C. Ngugi et al. presented a review of image processing techniques applied for plant leaf disease recognition [52]. They applied 10 DL models, videlicet, AlexNet, GoogLeNet, VGG-16, ResNet-101, DenseNet-201, Inception-v3, InceptionResNet-v2, Shuffle Net, SqueezeNet and Mobile-Nets on the Plant Village dataset. Based on the analysis of the performance of these models, the authors claimed that deep networks such as DenseNet-201 are most suitable for computation-intensive plant disease detection tasks. In contrast, shallow networks such as Shuffle Net and SqueezeNet are efficient for real-time mobile applications.

Kundu et al. [53] experimented with different deep learning models, videlicet, VGG16, VGG19, ResNet50, ResNet101, ResNet152, InceptionResNetV2, DenseNet121 on the publicly available dataset of the bell pepper plant. Based on the analysis of results, the authors claim that the ‘DenseNet’ model outperforms the above-stated models in predicting diseases in bell pepper. They also claimed that the model is less computation-intensive and can be adopted for real-time prediction [53].

Liu et al. presented the review of deep learning models employed for plant pest and disease prediction [54]. They highlighted the challenges in applying the deep learning models in plant disease prediction and highlighted the possible solutions for the identified challenges.

Amara et al. [21] performed experiments on 3700 images of banana leaves, a part of the PlantVillage dataset. The environmental conditions of illumination, size, background, pose, and orientation set for image capturing were different from conditions set by Mohanty et al. and Amara et al. who achieved the maximum classification accuracy of 96% by applying LeNet architecture. Yadav et al. [55] presented a deep learning model to identify disease areas, automatic segmentation, and bacteria from the peach leaf. Experimental evaluations on both lab data sets and actual cultivation reveal an overall classification accuracy of 98.75%. In [56], authors presented disease prediction from Rice leaves using transfer learning based on InceptionResNetV2. Chen et al. [57] also demonstrated the identification of Rice plant diseases using transfer learning. MobileNet-V2 was chosen for the backend, followed by an attention mechanism for learning inter-channel relationships. Comparison with other state-of-the-art public data sets, it presented an overall accuracy of 99.67%. Deep transfer learning is gaining tremendous popularity in plant leaf disease identification. In [58], the authors contributed an automatic disease identification method on the Vigna Mungo plant using three CNN architectures, videlicet, VirLeafNet-1, VirLeafNet-2, and VirLeafNet-3. Experimental evaluations on a self-made data set revealed 97.40% accuracy using VirLeafNet-3.

Wang et al. [18] performed a set of experiments on a small dataset of 552 images of apple leaves selected from the PlantVillage dataset. The dataset contains images of four stages of apple black rot disease. It includes 110 images of leaves of a healthy plant, 137 images of the early stage of the disease, 180 images of the middle stage, and 125 images of the late-stage black rot disease in apple leaves. The authors applied the VGG-16 model to the above-mentioned dataset. They fine-tuned the model through transfer learning. The model achieved the highest accuracy of 90.4%. The accuracy indicates the utility and effectiveness of the deep learning model, ‘VGG-16’, on a small training dataset for automatic estimation of plant disease severity [18]. Bhatt et al. applied the ResNet-50 model on 3750 images of tomato leaves from the PlantVillage dataset. They achieved an accuracy of 99.7% for the classification of leaf diseases in tomato plants [59].

Brahimi et al. [35] applied GoogLeNet architecture on 14,828 tomato leaves from the PlantVillage dataset. They achieved a maximum accuracy of 99.18%. Durmus et al. [40] applied the AlexNet model on the same dataset. They achieved an accuracy of 95.65%. Zhang et al. [60] applied the ResNet model on the dataset mentioned above and achieved an accuracy of 97.28%. These three experiments by researchers on the same dataset indicate that GoogLeNet architecture outperforms the AlexNet and ResNet models.

Researchers in [61] chose maize leaves for performing experiments. They designed their architecture of CNN and achieved an accuracy of 92.85% on a dataset containing 400 maize leaf images. Joly et al. [62] gathered the LifeCLEF dataset. Its training dataset contains 91,758 images, and the testing dataset contains 21,446 images of various species of plants. Ghazi et al. [63] applied three deep learning architectures, namely, GoogLeNet, AlexNet, and VGGNet, on the LifeCLEF dataset for plant identification. They achieved the highest accuracy of 80.18% by combining the GoogLeNet and VGGNet models using the score-based fusion technique. In this hybrid architecture, at the first layer, tuning weights is completed to extract features such as edges or color blobs. At higher layers, specific patterns are extracted that are observed in plant organs [63].

Ferentinos [24] used an open database of 87,848 leaf images for performing a set of experiments. The images were captured from healthy as well as diseased plants. The dataset includes 58 different classes and 25 species of peach, pepper, pumpkin, raspberry, soybean, etc. Its training dataset contains 70,300, and the testing dataset contains 17,548 images. The author applied five different models to this dataset. They achieved an accuracy of 99.06% by using AlexNet, 99.49% by applying AlexNetOWTBn, 92.27% by using GoogLeNet, 98.96% by applying Over feat, and 99.53% accuracy by applying the VGG model. The authors performed a set of experiments on original images and pre-processed images. All models yielded higher accuracy on original images than pre-processed images. However, a significant reduction in computation time is noticed on applying the above-stated models on pre-processed, down-scaled, and squared images [24].

Sladojevic et al. [25] downloaded 4483 images from the Internet for creating a database. These images are categorized into 15 classes. A total of 13 classes represent diseased plants, 1 class represents healthy leaves, and 1 class represents a background. The experimental results show an overall accuracy of 96.3%.

Authors in [20] performed experiments on images of leaves of watermelon, orange, corn, grapes, cherry, and blueberry, with a dataset size of 54,306 images from the PlantVillage. They achieved an accuracy of 82% by applying VGG16 architecture, 98% accuracy by using Inception-V4, 99.6% by ResNet50, 99.6% by ResNet101, 99.7% by ResNet152, and 99.75% accuracy by using DenseNet121 CNN architecture.

Authors in [38,64] captured authentic images from cultivated fields. They used these images to develop an automatic plant disease detection system. DeChant et al. [64] used the computational pipeline of CNNs. The first CNN model is trained for classifying small regions of images based on the presence of disease lesions on leaves. The predictions about the presence of lesions were fed to another CNN model in the pipeline trained for classifying an entire image. Researchers in [23] use images processed in a laboratory with controlled lighting, illumination, and intensity. Segmentation on leaves was applied, and background removal was performed. The researcher in [65] applied the GoogLeNet model on the Expanded dataset (XDB). This dataset contains original images. Researchers successfully identified 79 diseases of 14 different plant species through individual lesion and spot identification.

**Figure 4:**
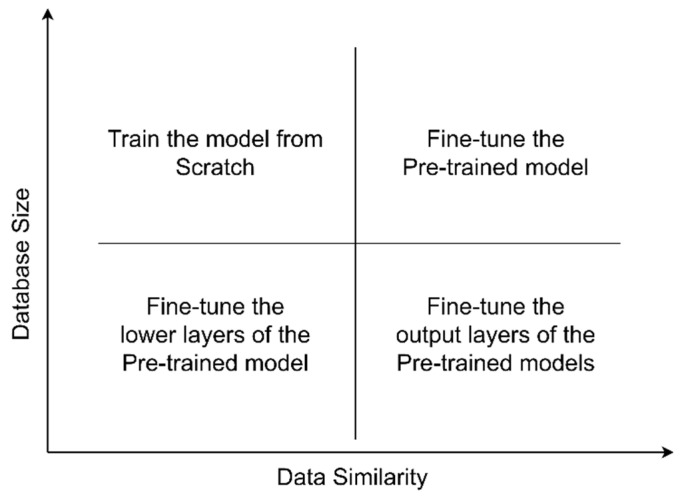
Approaches in transfer learning.

Deep learning models require massive datasets for training. Transfer learning alleviates the problem of insufficient training datasets. In the case of the availability of small datasets, transfer learning outperforms the models trained from scratch. Therefore, researchers use pre-trained models. Deng et al. applied the ImageNet model [66], and Everingham et al. used Pascal VOC [67] datasets to pre-train deep learning architectures. These researchers employed the concept of transfer learning and used pre-trained models for classification. Figure 4 shows different ways to apply transfer learning. Pre-trained models are applied as a feature extractor in case of the small size of the dataset and high data similarity with a pre-trained model. These models obtain features with their learned weights. There is a need to fine-tune the weights of a network and train it on a new dataset when the size of the dataset is large. Moreover, there is a need to modify the number of nodes at the final layer of the network, according to the number of different classes in a problem [63].

Khan et al. [27] applied contrast stretching and segmentation of disease areas before classifying diseases identified in apple and banana leaves. Next, they focus on determining the severity of black rot disease on apple leaves. Finally, they also classified leaves into four stages of the disease: healthy, early, middle, and end-stage. Researchers divide datasets into different ratios of training and testing to evaluate the efficacy of deep learning models. Exemplified as 60:40, 80:20 and 70:30, etc. Researchers in [68] divide the dataset into three parts: 80% training, 10% validation, and 10% testing to minimize the bias.

Deep learning models have a strong advantage over feature learning. These models automatically extract features from images. However, they require large datasets to enhance classification accuracy [69]. In the case of insufficient datasets, researchers use pre-trained models. As per details given in [33], deeper networks are more efficient in training and provides better results. Strategies such as skip connections [32], optimization methods [70], transfer learning [69], initialization strategies [71], layer-wise training [72], and Batch Normalization [19] effectively deal with problems such as vanishing gradients, degradation, and internal covariate shift for deep neural networks.

### 3.4. Common CNN Architectures

The evolution of CNN began with LeNet [73] in 1998. It became popular in 2012, when AlexNet won the ‘ImageNet Large Scale Visual Recognition Challenge [74] with great distinction. Further improvements in architectures increase the accuracy rate and decrease the error rate. Applications of CNN architectures are extended in the field of image classification. Each architecture has unique advantages and characteristics which make it suitable for a specific data set and experimental conditions [75]. Commonly used architectures for image classification are given as follows.

#### 3.4.1. LeNet-5

**Figure 5:**
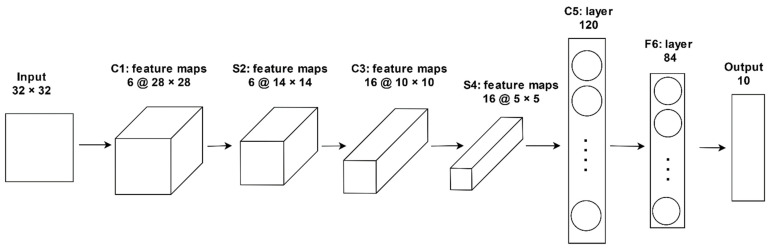
LeNet-5 architecture [73].

LeNet-5 is a pioneering, seven-level, mostly straightforward architecture, as shown in Figure 5. It contains three convolutional layers (C1, C3, and C5), two pooling layers (S2 and S4), one fully connected layer (F6), and an output layer. Its details are described in [75]. Initially, LeNet-5 architecture was used for recognizing handwritten digits from an image of dimensions 32 × 32 × 1. A convolution was performed by using six filters of dimensions 5 × 5 and a stride of 1. A feature map of dimensions 28 × 28 × 6 is obtained. It uses an average pooling with the same filter width and stride 2. It reduced the dimensions to 14 × 14 × 6. Another convolutional layer was used with 16 filters of dimensions 5 × 5 and a stride of 1. It gave a feature map of dimension 10 × 10 × 16, a pooling layer with a filter size of 2 × 2, and a stride of two gave a feature map of dimensions 5 × 5 × 16. The next convolutional layer, C5, contains 120 feature maps. The 400 nodes of the previous layer with dimensions (5 × 5 × 16) are connected to 120 neurons. Layer F6 contains 84 units. It is fully connected with 120 units of convolutional layer, C5. The output layer comprises Euclidean RBF (Radial Basis Function) units for each class with 84 inputs. The output of each RBF unit is computed as per the formula given in Equation (3). Here, $y_{i}$ represents the output of the *i*th RBF unit, $x_{j}$ represents *j*th unit of an input vector, and $w_{ij}$ represents the value of a parameter vector at *ij* position.

(3) $$y_{i}=\overset{\;}{\underset{j}{\sum }}{\left(x_{j}-w_{ij}\right)}^{2}$$

As per the discussion given in [76], LeNet is faster to deploy, acquiescent to parallel hardware, and efficient in performing small-scale image recognition problems.

The basic architecture of LeNet is applied for the classification of two types of diseases, namely Black Sigatoka and Banana Speckle in leaves of banana. It achieved accuracy in the range of 92–99% under challenging images capturing such as complex backgrounds, different sizes, low illumination, and improper orientation [21]. As discussed in [28], a modification is completed in LeNet for detecting quick decline syndrome in olive and achieved an accuracy of 99%. Its comparison with three feature vector representations, videlicet, SIFT, Local Binary Patterns, and Gabor filter, prove its better effectiveness. Architecture with three convolutional layers, two pooling layers, and one fully connected layer similar to LeNet-5 is applied to images of infected leaves of cucumber for detecting diseases. It yielded better results than classifiers, namely, Support-Vector Machines (SVM), Random Forest, and AlexNet [37]. Kerkech et al. also used LeNet-5 on Unmanned Aerial Vehicle Red Green Blue (UAV RGB) images for detecting Esca disease in the vineyard. It gave four categories, namely, ground, healthy, potentially diseased, and diseased class. It achieved a classification accuracy of 95.80% [77]. However, the LeNet model requires less storage space and short training time, but it suffers from inadequate training. Employing the ReLU activation function causes the problem of the vanishing gradient. Thus, it reports low prediction accuracy and becomes unsuitable for plant disease prediction.

#### 3.4.2. AlexNet

AlexNet architecture came into existence after showing its outstanding performance in ImageNet Large Scale Visual Recognition Challenge-2012. This architecture is similar to LeNet, but it is deeper and uses about 60 million parameters. As per the discussion given in [72], it achieved the top-five error of 15.3%, lower than 26.2% achieved by the second-best entry. The architecture of AlexNet consists of five convolutional and three fully connected layers. It uses the ReLU activation function. It uses the MaxPooling layer, local response normalization, and many GPUs for training a network. The first convolutional layer filters an input image of size 224 × 224 × 3 through 96 kernels of dimensions 11 × 11 × 3 by a stride of 4. The second convolutional layer receives a normalized response and pooling output of the first layer. It applied filters by using 256 kernels of dimensions 5 × 5 × 48. The next three layers are connected to each other without using any normalization or pooling layer. The third convolutional layer contains 384 kernels with dimensions 3 × 3 × 256. In the fourth convolutional layer, 384 kernels of dimensions 3 × 3 × 192 are used. In the fifth convolutional layer, 256 kernels of dimensions 3 × 3 × 192 are used. Each fully connected layer contains 4096 neurons. The output of the last fully connected layer is fed to a softmax. It yields 1000 class labels. The ReLU layer followed all the five convolutional layers. ReLU nonlinearity is followed by local response normalization. These normalization layers are added to help generalization. The response-normalized activity is defined as given in Equation (4). Here *b* is the regularized output for kernel *i* at position (*x*, *y*). The activity of a neuron is calculated by applying ith kernel at (*x*, *y*) position. *N* denotes the total number of kernels in a layer. The constants *α*, *β*, *k*, and *n*, are the hyper-parameters, and their values are determined by using a validation set.

(4) $$b_{xy}^{i}=a_{xy}^{i}/{\left(k+\alpha \;\overset{min\left(N-1,i+n/2\right)}{\underset{j=max\left(0,i-n/2\right)}{\sum }}\;{\left(a_{xy}^{j}\right)}^{2}\right)}^{\beta }$$

AlexNet reduces the problem of overfitting in fully connected layers. Therefore, it leads to a dropout of the regularization method. The regularization method starts learning the excessive details of the dataset, including inaccurate data entries and noise. Thus, it faces the problem of overfitting when a model is trained with a vast dataset. In such cases, a model fits well with the training dataset but may fail to categorize new data entries correctly. However, the AlexNet model reports higher accuracy than LeNet but dealing with the imbalanced dataset and fine-tuning of the model according to the type of dataset still presents unresolved issues.

#### 3.4.3. VGGNet

**Figure 6:**
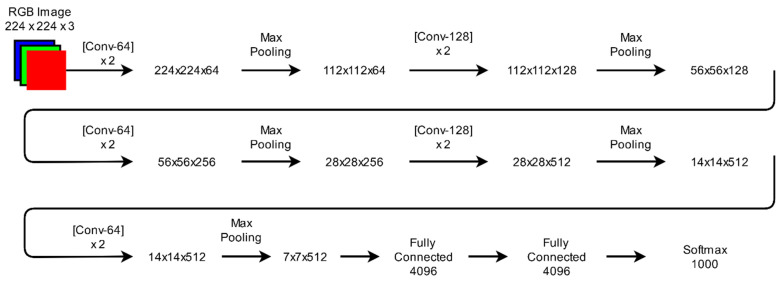
VGG-16 architecture [31].

VGGNet [31] is devised by the Visual Geometry Group from the University of Oxford. It was the first runner-up in the ILSVRC-2014 challenge. The localization and classification accuracy of this model increases with an increase in the depth of a model. VGGNet is a simpler network. It uses small convolutional filters of dimensions 3 × 3 with a stride of one in all layers. It includes a max-pooling layer of dimension 2 × 2 with a stride of two. The architecture of VGGNet is shown in Figure 6. It receives an RGB image of dimension 224 × 224 × 3 as an input. In the training dataset, the mean average value of RGB is subtracted from each pixel to perform pre-processing. A pre-processed image is passed through a stack of convolutional layers followed by five max-pooling layers. It uses the first two fully connected layers with 4096 channels in each layer and the third fully connected layer with 1000 channels. The last layer performs a 1000-way classification. At the last stage, a softmax layer works to determine multi-class probabilities.

#### 3.4.4. GoogLeNet

**Figure 7:**
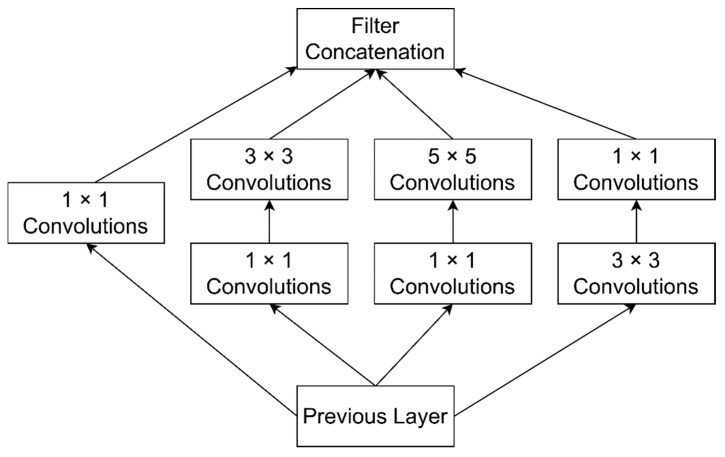
Inception module with dimension reduction [79].

GoogLeNet [78] is inspired by the Inception module [79], which is the winner of ILSVRC-2014. It is a deeper and broader architecture. It consists of 22 layers with 224 × 224 receptive fields and very small convolutions of 1 × 1, 3 × 3, 5 × 5. GoogLeNet uses 1 × 1, 3 × 3, 5 × 5 convolutions and 3 × 3 max-pooling layers together to extract different kinds of features. It has nine linearly stacked inception modules. The Inception module is a combination of 1 × 1, 3 × 3, 5 × 5 convolutional layers with their outputs concatenated into a solitary output vector. This creates an input for the next layer, as shown in Figure 7. GoogLeNet makes two significant modifications to an original inception module. First, a 1 × 1 convolutional layer is applied before other layers. Second, it uses a parallel max-pooling layer. GoogLeNet reduces dimensions which further reduces the computation cost. At the end of the last inception module, it uses global average pooling rather than fully connected layers. As there is an increase in the number of layers and the number of neurons in each layer, networks become more prone to overfitting. GoogLeNet uses sparsely connected network architectures rather than fully connected networks, specifically, inside convolutional layers.

GoogLeNet provides a good solution for overfitting problems and reduces computational and storage costs.

#### 3.4.5. ResNet

**Figure 8:**
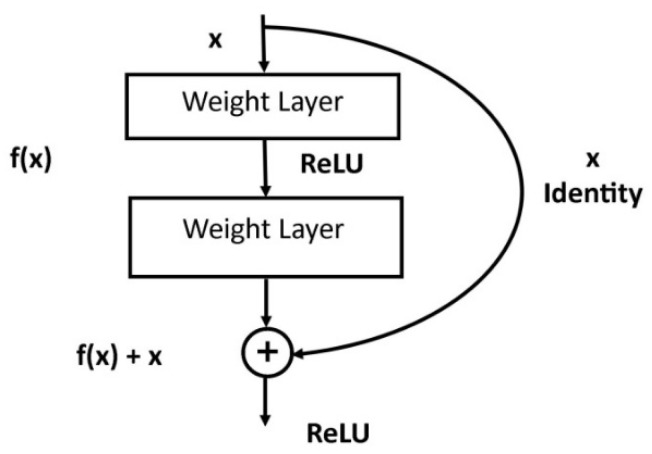
Building block of residual learning [80].

Kaiming et al. developed ResNet. It was the winner of ILSRVC-2015 with a top-five error rate of 3.57% [32]. This deep residual learning framework is motivated by the degradation problem due to more and more layers to a network. It becomes difficult to train a deep network due to the vanishing gradient problem. During backpropagation, gradients of loss are calculated concerning weights. The gradients tend to become smaller on moving backward in a network. Thus, the performance of the network saturates or degrades. This indicates that lower layers are slow learners than upper layers of a network. Another problem of deeper networks is performing optimization on large parameter space. Therefore, adding more layers causes higher training errors. ResNet builds the network through residual modules for the training of deep networks, as shown in Figure 8.

ResNet-50 is a convolutional network that contains residual blocks with 50 convolutional layers. It includes about 25.6 million parameters. In ResNet-101, the number of parameters is increased to 44.5 million. In ResNet-152, the number of parameters are 60.2 million [80].

#### 3.4.6. ResNeXt

This architecture is an extension of ResNet. Here, ResNet blocks are replaced by ResNeXt blocks based on the strategy of ‘split-transform-merge.’ ResNet does not create filters for the entire channel depth of an input. It splits the input into groups. Each group represents a channel. As per experiments completed in [81], increasing the cardinality of a network makes more improvement in the performance of a model than an increase in the depth of a network. Applying ResNeXt architecture with a faster R-CNN detector is effective in disease detection and pest recognition in tomato leaves [82].

#### 3.4.7. DenseNet

**Figure 9:**
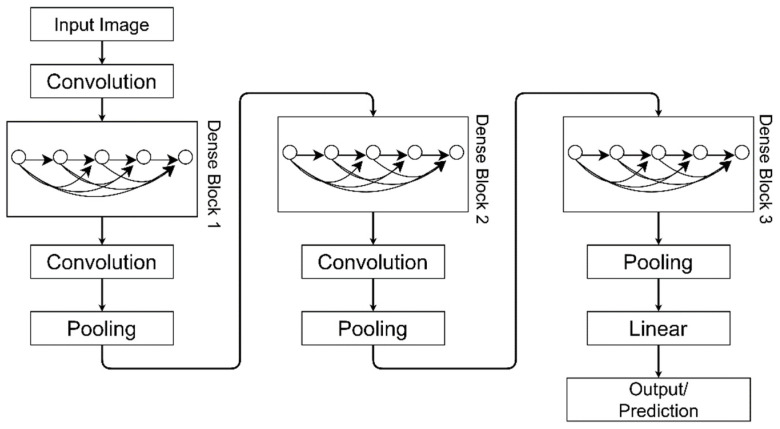
Architecture of DenseNet containing 3 Dense Blocks [33].

G.Huang et al. proposed DenseNet, a densely connected network. It consists of two modules, namely, Dense Blocks and Transition Layers, as shown in Figure 9. In this network, all layers are connected directly with each other in a Feed-Forward manner. A DenseNet of N layers contains N(N + 1)/2 direct connections [33]. Each layer receives feature maps of previous layers as inputs. A Dense Block is composed of Batch Normalization, ReLU activation, and 3 × 3 Convolution. Transition layers lie between two Dense Blocks. These are made up of Batch Normalization, 1 × 1 Convolution, and average pooling. To concatenate all feature maps in each dense block, feature maps of all layers are of the same size.

#### 3.4.8. SqueezeNet

**Figure 10:**
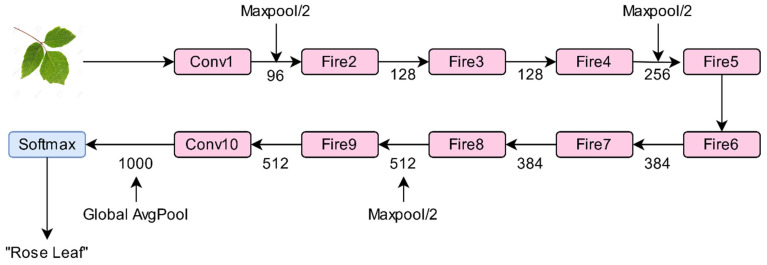
Architecture of SqueezeNet [83].

SqueezeNet [83] is a sandwich of eight fire modules between two convolutional layers, as shown in Figure 10. The fire module consists of a squeeze convolutional layer with a filter of dimensions 1 × 1. It is fed to an expanded layer. This layer contains a mixture of 1 × 1 and 3 × 3 convolutional filters. For each fire module, the number of filters gradually increases from beginning to end of a network. Max-pooling is performed after convolution1, fire4, and fire8 layers with a stride of two. It makes use of the ReLU activation function. It uses Dropout after module Fire9.

Some researchers developed their architectures inspired by the above-discussed architectures. The details of these architectures are given below:

#### 3.4.9. LeafNet

J. Chen et al. developed a CNN model, LeafNet, to identify diseases in tea leaves [84]. This model is an improvement over AlexNet. It is built by reducing the number of filters in the convolutional layer and the nodes in the fully connected layer. This effective reduction in the number of network parameters helps in reducing the problem of overfitting.

#### 3.4.10. M-bCNN

Lin et al. proposed a unified CNN model, matrix-based CNN (M-bCNN), to detect diseases in wheat leaves using image classification. The model outperforms VGG-16 and AlexNet due to the substantial increase of data streams, link channels, and neurons at a fair growth of computational requirements [85]. Liang et al. proposed two CNN architectures for the recognition of rice blast diseases [86]. These are similar to LeNet-5. The first CNN comprises four convolutional layers with four max-pooling layers and three fully connected layers. In this network, ReLU is added after each layer. The second CNN has the same convolutional and max-pooling layers as in the first CNN but has only two fully connected layers. To avoid the problem of overfitting, dropout layers are added in both networks.

#### 3.4.11. Comparison of Common CNN Architectures

Deep learning is a powerful technique. It makes use of ANNs containing a larger number of processing layers than traditional Neural Network methodologies [24]. Convolutional Neural Network (CNN) is one of the most preferred deep learning techniques. It automatically learns important features from raw data. This network extracts the color and texture features of an image for the identification and classification of images. It is applied for identifying objects [87], faces [88,89,90], bones [91], handwritten digits [92], traffic signs [93], etc. Besides, CNNs are successfully used in the field of agriculture for recognition of plant species [63], yield management [94,95], weed detection [96], soil and water management [97], fruit counting [98], diseases and pest detection [82,99], evaluating the nutrient status of plants [100], and monitoring of fields [101].

In this manuscript, the authors focus on the application of CNN in plant disease identification and classification. An extensive literature survey shows that researchers use CNN for disease localization, identification, and classification. The work discussed in [35,38,81,101] focuses on disease localization, identification. Researchers in [18,26,28,64,77,86,102] worked on detection of a single disease in plants. Researchers in [17,22,23,24,25,27,35,37,39,40,59,60,61,65,68,82,84,85,103,104,105,106] make use of CNN for identifying multiple diseases in plants. The model proposed in [18] identifies the severity of plant diseases. However, identifying the severity of plant disease is more challenging than the classification of plant diseases. This is due to the presence of intraclass similarities with interclass variance.

AlexNet is applied to images of plant leaves for identifying 26 diseases in 14 species of crops. For example, researchers in [23] achieved an accuracy of 99.27% for diseases such as Apple Scab, Apple Black Rot, Apple Cedar Rust, Cherry Powdery Mildew, Corn Gray Leaf Spot, Corn Common Rust, Corn Northern Leaf Blight, Grape Black Rot, Grape Black Measles (Esca), Grape Leaf Blight, Orange Huanglongbing, Peach Bacterial Spot, Bell Pepper Bacterial Spot, Potato Early Blight, Potato Late Blight, Squash Powdery Mildew, Strawberry Leaf Scorch, Tomato Bacterial Spot, Tomato Early Blight, Tomato Late Blight, Tomato Leaf Mold, Tomato Septoria Leaf Spot, Tomato Two Spotted Spider Mite, Tomato Target Spot, Tomato Mosaic Virus, and Tomato Yellow Leaf Curl Virus.

Brahimi et al. achieved an accuracy of 98.66% for classifying nine diseases of tomato plants [35]. Authors applied visualization methods for localizing leaf disease regions for understanding the symptoms of a disease. Ferentinos [24] applied CNN to detect and diagnose plant diseases from an open dataset of 87,848 images. The dataset contains 25 different species of plants and 58 distinct categories of plants’ diseases. Experimental results claim that the success rate of VGGNet is 99.53%, with a learning rate ranging from 0.01 to 0.0001. The learning rate is decreased by 1/2 and 1/5 alternatively on every 20 epochs. Durmus et al. [40], in their work, applied two deep learning models, AlexNet and SqueezeNet, for detecting leaf diseases of tomato plants. Research reveals that AlexNet architecture performed better than small-sized architecture sequeezeNet. A comparison of three robust architectures AlexNet, GoogLeNet, and VGGNet, is performed on LifeCLEF 2015 dataset for automated plant identification [61].

Fine-tuning improves the performance of pre-trained architectures, namely, GoogLeNet and VGGNet. Thus, the GoogLeNet and VGGNet provide better results than the AlexNet model. A comparison of AlexNet with GoogleNet shows that GoogleNet reduces the number of parameters to 4 million from 60 million parameters as used in AlexNet [29]. Experimental results obtained on applying AlexNet and GoogLeNet to classify tomato diseases and symptoms visualization [35] show that GoogLeNet provides better accuracy of 99.19% than AlexNet with 98.66% accuracy. GoogLeNet architecture increases nonlinearity without outbursts in the number of weights. Hence, it becomes superior in performance to AlexNet. A comparison of AlexNet and GoogLeNet presented by Mohanty et al. in [23] on PlantVillage also claims that GoogLeNet architecture outperforms with an accuracy of 99.35%. GoogLeNet architecture achieved an accuracy of 94% to identify plant diseases using individual lesions [65].

Further developments lead to the release of new versions of GoogLeNet. The successive versions of GoogLeNet are named InceptionvN. Here, N is a version number [29]. Inception v3 improves the performance of a network with a low computational cost. It is favorable for applications where computational power and memory are limited. The experimental results obtained in [104] show that Inception v3 is a robust network for detecting cassava diseases using transfer learning that achieved an overall accuracy of 93%. One more version of GoogLeNet, Inception v4, replaces the filter concatenation stage. It unites residual connections with Inception architecture to speed up the training of Inception networks. The accuracy acquired by this model is 98% [20].

A comparative analysis of VGGNet, Inception-v3, and ResNet-50 architectures for estimating the severity of plant disease [18] shows that VGG-16 outperforms VGG-19 with an accuracy rate of 90.4%. This comparative study also revealed that the performance on small datasets could be improved through pre-trained deep models by fine-tuning a network. VGG-16 is less effective than Inception-v4, ResNet (with 50, 101, and 152 layers), and DenseNet in the case of identification of plant diseases [20]. This is due to the use of a larger number of parameters in VGGNet than the number of parameters in deeper networks such as DenseNets, ResNet, and Inception-v4. A comparison of VGGNet with GoogLeNet shows that GoogLeNet trains faster than VGGNet. Moreover, the size of a pre-trained GoogLeNet is smaller than VGGNet.

A comparison of CNN models given in [59] for the health assessment of crops shows that the accuracy of ResNet-50 is more than Xception, Inception-v3, and VGG-19. This is due to the depth of the network and the correction of residual errors. The work in [20] shows that fine-tuned networks such as ResNet-50, ResNet-101, and ResNet-152 perform better than VGG-16 and Inception-V4. Picon et al. perform an extension of work given in [107]. They applied ResNet-50 on images captured by mobile for detecting multiple crop diseases in a real-time environment. The model achieved a classification accuracy of 96% in the identification of plant diseases [68]. Another network model, DenseNet, is similar to ResNet. However, it concatenates feature maps, whereas ResNet performs a summation of feature maps. DenseNet uses a skip connection from each previous layer, but ResNet uses a skip connection from one previous layer. DenseNet’s advantageous as it reduces the vanishing-gradient problem and requires a fewer parameters. Irrespective of the increasing number of epochs, it continually improves accuracy. It does not face the problem of performance degradation and overfitting. DenseNet, with 121 layers applied for identification of plant disease, achieved the highest accuracy of 99.75% among models, namely, VGG, Inception, and ResNet [20]. SqueezeNet [83] is built on three design strategies. First, it reduces filter size from 3 × 3 to 1 × 1, leading to a nine times reduction in the number of parameters. Second, it decreases the number of input channels using squeeze layers, and its delay down-sampling, leading to large activation maps in convolutional layers. Durmus et al. [40] applied SqueezeNet for detecting diseases in tomato leaves. SqueezeNet is a lightweight model of 2.9 MB. It is approximately 80 times smaller than AlexNet and achieves the same level of accuracy. Its smaller size makes it useful for mobile-based disease detection applications. Ha et al. applied the VGG-A model (Visual Geometry Group-Architecture), a network of eight convolutional layers and two fully connected layers on input images of dimensions 200 × 200 to distinguish Fusarium wilt diseased radish from healthy radish [26]. The model achieved an accuracy of 93.3%. Oppenheim and Shani applied the VGG model of eight learnable layers with five convolutional layers and three fully connected layers to classify potato disease [22]. The classification accuracy of the VGG model is dependent on the size of the training dataset. It yielded classification accuracy ranging from 83% to 96% in the case of potato disease classification.

**Figure 11:**
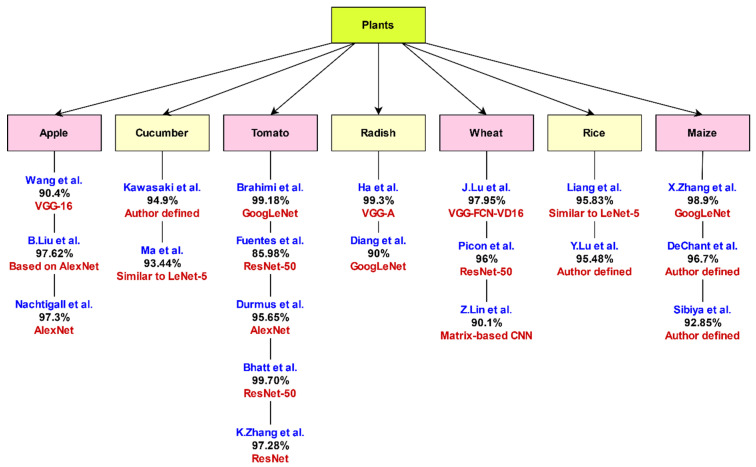
Comparison of CNN architectures based on plant types and accuracy.

Discussion of common Deep Convolutional Neural Network (DCNN) architectures shows that the selection of suitable CNN model depends on the type of dataset, size of the dataset, and experimental conditions. In Table 3, the authors present a comparison of architectures of standard CNN models used to detect diseases in plant leaves. Figure 11 demonstrates a comparison of different architectures based on plant type and accuracy achieved by different authors.

On comparing the accuracy of machine learning models and Deep Convolutional Neural Networks models applied on different sizes of datasets for detection of plant diseases using images of leaves, it is observed that Deep Convolutional Neural Networks outperform machine learning models [64]. Moreover, deep learning bypasses the handcrafted features. Instead, it automates and optimizes feature extraction, the availability of more data, and powerful computation engines such as GPUs help overtake deep learning models.

**Figure 12:**
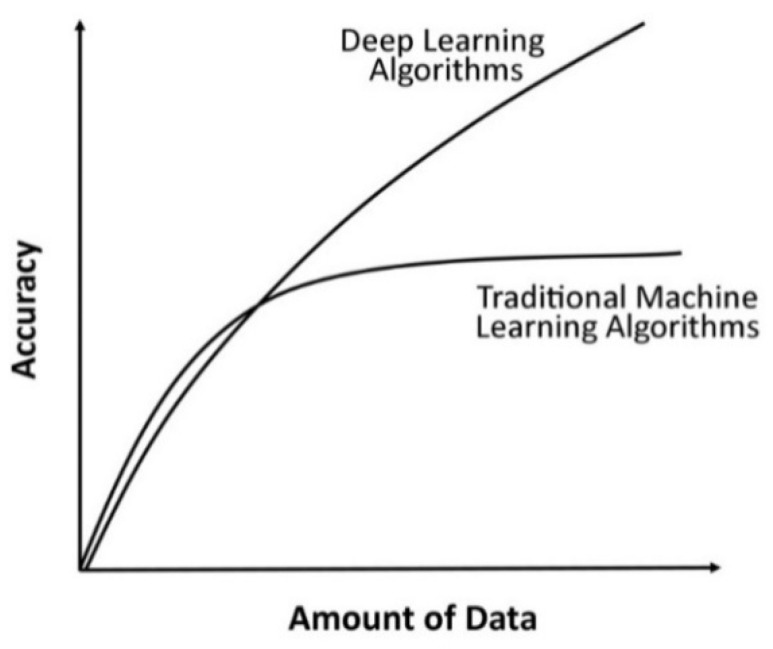
Comparison of classification accuracy of machine learning and deep learning models [64].

Due to outstanding performances, deep learning models gain popularity over machine learning models. The comparison curves are shown in Figure 12. The X-axis refers to the amount of data or size of a dataset, and the Y-axis refers to the accuracy achieved by different machine learning and DCNN models.

### 3.5. Optimization Techniques

There is a vital requirement of applying a suitable optimization technique to improve the effectiveness of a CNN model. The discussion in Section 3.5.1, Section 3.5.2, Section 3.5.3, Section 3.5.4 and Section 3.5.5 gives a brief description of the most applied optimization techniques. The authors present a comparison of the most commonly used optimization techniques in Table 4.

#### 3.5.1. Batch Gradient Descent (BGD) Optimization

In this technique, a complete training dataset is scanned for updating the parameter *x,* as given in Equation (5). Gradient calculation takes a long time to scan millions or billions of samples in a training dataset. Moreover, it is quite difficult to feed all samples to a model simultaneously due to limited computational memory. As claimed in [69], the drawbacks make Batch Gradient Descent non-preferable for a deep learning model to update the parameters. Here, $x_{k+1}$ is the updated parameter of a model, $x_{k}$ is the parameter of a model at *k*th iteration, $t_{k}$ is the step size for *k*th iteration, and Δf(*x_k_*) is a loss function based on the training data instance indexed by *k*, for iteration 1 to *n.*

(5) $$x_{k+1}=x_{k}-t_{k}\Delta f\left(x_{k}\right){\;}^{\left(1:n\right)}\;$$

#### 3.5.2. Stochastic Gradient Descent (SGD) Algorithm

The Stochastic Gradient Descent algorithm updates the parameters of a model in a negative direction. It calculates the gradient and updates the parameters of a model for each training sample. It brings randomness by calculating the cost of a single data point and the corresponding gradient instead of the computing cost of all the data points. This quickly updates the steps and reaches the minimum in small time duration. Therefore, it is one of the most commonly used optimization algorithms. A small step size helps SGD converge to a good point, making the training process slow. In addition, when GPUs are used to conduct the calculations, the efficiency is reduced by frequent commutation of data between the GPU memory and the local memory. The update occurs as shown in Equation (6). Here, $x_{k+1}$ is the updated parameters of the model, $x_{k}$ is the parameters of the model at iteration *k*, $t_{k}$ is the step size for iteration *k*, Δf(*x_k_*) is a loss function based on the training data instance indexed by *k*, and *i* is the iteration index.

(6) $$x_{k+1}=x_{k}-t_{k}\Delta f{\left(x_{k}\right)}^{i}\;$$

#### 3.5.3. AdaGrad

AdaGrad is an optimization technique with parameter-specific learning rates. These learning rates are adjusted based on how frequently a parameter is updated during training. It performs more minor updates or low learning rates for the parameters with frequently occurring features. It performs more significant updates or high learning rates for parameters with infrequent features. This makes Adagrad suitable for dealing with sparse data.

#### 3.5.4. Root Mean Square Propagation (RMSprop)

The objective of RMSprop is to reduce the number of oscillations without adjusting the learning rate. RMSprop is used when there is a high fluctuation in gradients of successive mini-batches. In such cases, the weights are finely adjusted by dividing the learning rate by the average exponential decay of squared gradients. RMSprop automatically adjusts the learning rates. It uses different learning rates for the different parameters. The RMSprop optimizer is similar to the gradient descent algorithm with momentum. However, it differs in the mechanism of calculation of gradients. It limits the oscillations in the vertical direction. Therefore, the algorithm takes more giant steps in the horizontal direction and converges faster.

#### 3.5.5. Adaptive Moment Estimation (Adam) Optimizer

As given in [109], the Adam Optimizer computes the average and square of a gradient for each parameter. The Adam optimization algorithm is a blend of gradient descent with momentum and RMSprop algorithm. Like RMSprop, it uses the squared gradients to scale the learning rate. However, it takes advantage of SGD with momentum by using the moving average of the gradient instead of the gradient itself. The main advantage of this optimizer is that it requires low memory and usually works well even with a slight tuning of hyper-parameters.

Wang et al. used the SGD optimization algorithm in VGG16, VGG19, Inception-V3, and ResNet50 models in both training from scratch and fine-tuned models. The SGD optimizer results in poor generalization and leads to a local optimization while using ResNet architecture. In addition, it puts residual mapping in building blocks of ResNet to zero too early. The best accuracy of 90.4% is achieved by the VGG16 model with transfer learning [18].

K. Zhang et al. applied SGD and Adam optimization on AlexNet, GoogLeNet, and ResNet architectures individually. The comparison shows that the performance of the SGD optimization method is better than Adam optimization in all networks. ResNet with the SGD optimization algorithm attains the highest accuracy of 96.51%, as claimed in [60]. Bhatt et al. applied SGD, Adam Optimizer, and RMSProp optimizers on VGG-19, Inception-v3, Xception, and ResNet-50 architectures as given in [59] to update weights of the parameters to minimize the cross-entropy loss. SGD faces the optimum local problem; thus, Liu et al. preferred Nesterov’s Accelerated Gradient for better performance [39].

### 3.6. Frameworks

Developments in Artificial Intelligence provide various tools and platforms to apply deep learning in different application areas. A brief description of commonly used frameworks is given in Section 3.6.1, Section 3.6.2, Section 3.6.3, Section 3.6.4, Section 3.6.5, Section 3.6.6, Section 3.6.7, Section 3.6.8 and Section 3.6.9.

#### 3.6.1. TensorFlow

As per the discussion given in [110], TensorFlow is the commonly used framework for deep learning applications. It is a popular open-source software library. It is written in C++ and Python. Google develops the library for artificial Neural Networks. It helps perform numerical computations on a CPU, GPU, server, desktop, or mobile, by using data flow graphs. Ramcharan et al. applied TensorFlow for implementing an image recognition code for the detection of cassava disease [104]. Picon et al. used TensorFlow for deploying mobile applications for the classification of crop diseases [68]. Authors in [59] used TensorFlow in the backend with Keras Deep Learning framework for assessing the health of crops.

#### 3.6.2. Theano

Theano [111,112] is a powerful Python library. It allows us to define, evaluate and optimize numerical operations, including multidimensional arrays with a higher level of efficiency [113]. This tool includes integration with NumPy, transparent use of a GPU, generation of dynamic C code, speed optimization, and efficient symbolic differentiation. It is not easy to understand the syntaxes of Theano. However, it is still in common use due to its advantage of highly optimized performance. Researchers make use of Theano in the backend with Keras or with other Deep Learning libraries. Exemplified as researchers in [38] used the Theano framework by running their code on a Geforce GTX 1080 GPU for automatic disease diagnosis in a wheat field. Wang et al. performed experiments on an Ubuntu workstation with a CPU of Intel Core i5 6500 and a GeForce GTX TITAN X GPU to automatically estimate the severity of diseases from the image dataset. The technique yields an accuracy of 90.4% [18].

#### 3.6.3. Keras

Keras is an open-source, simple, and easy-to-use library for implementing deep learning. It is an application programmer’s interface written in Python. It serves the purpose of quick experimentation and uses the same code for both CPU and GPU. It can run on top of TensorFlow [30] and Microsoft cognitive toolkit. Tools such as Caffe and Theano incorporate popular architecture, namely, VGGNet, AlexNet, GoogLeNet, in the form of classes or libraries.

#### 3.6.4. Caffe

Caffe [114] is an open-source deep learning framework developed by Yangqing Jia at UC Berkley. It provides a good interface in Matlab, C++, and Python. It is very fast and efficient; thus, it is helpful in building Convolutional Neural Networks for image classification. This tool allows applying Neural Networks without writing code. It is the most accessible framework to evaluate the performance of deep architectures [115]. Ha et al. used the Caffe framework for training a CNN model to classify Fusarium wilt of radish with an NVIDIA DIGITS 5 toolbox [26]. They used an Intel processor Core i7-5930K with three NVIDIA Titan X 12 GB GPUs and four 3072 Cuda cores in the Linux platform, Ubuntu 14.04. Caffe provides ease of use for evaluating the performance of standard deep architectures than other frameworks such as Theano and Torch; it is used for implementing CNN-based models for plant disease identification as discussed in [35,36,39]. The Caffe model is accelerated by integration with the cuDNN library [116].

#### 3.6.5. Torch

Torch7, based on the Lua programming language, is an open-source library for numerical and scientific operations. This tool provides efficient GPU support, routines for indexing, transposing and slicing, n-dimensional array, linear algebra routines, etc. Ferentinos [24] used the Torch7 framework for comparing the performance of different CNN models for plant disease detection and diagnosis. They implemented it on an NVIDIA GTX1080 GPU. Facebook developed PyTorch, a framework similar to Torch. This is one of the most flexible frameworks. Its features such as easy-to-use API, support of Python language, dynamic computation graphs increase its popularity.

#### 3.6.6. Neuroph

This framework is an integrated environment. It consists of a Java Neural Network library, Java IDE based on NetBeans Platform for creating and deploying Neural Networks. This framework is suitable for building a CNN model without much knowledge of programming languages, such as C, Python, and MATLAB [61].

#### 3.6.7. Deeplearning4j

Deeplearning4j is an open-source, distributed deep-learning library written in Java. It can be used with API languages such as Scala, Python, Kotlin, and Clojure. For image recognition, Deeplearning4j is as fast as Caffe using multiple GPUs. Furthermore, if Deeplearning4j is created with Keras, they can import models from Tensorflow and other Python frameworks. For example, Amara et al. used the deeplearning4j framework to evaluate the performance of their model for classifying banana leaf diseases [21].

#### 3.6.8. Pylearn2

As per the literature study, there is minimal use of Pylearn2 because it is ineffective in solving large-scale problems.

#### 3.6.9. DL MATLAB Toolbox

Deep learning MATLAB Toolbox provides a framework for designing and implementing deep neural networks. This framework is written in MATLAB, Java, C, C++ and supported on Linux, macOS, and Windows platforms. In addition, the toolbox supports transfer learning with a library of pre-trained models includes SqueezeNet, Inception-v3, and ResNet-101.

The above discussion highlights the details about different CNN frameworks available in the literature. The authors present a comparative analysis of existing frameworks in Table 5.

### 3.7. Analysis of DCNNs for Plant Leaf Disease Identification

The researcher has presented plenty of plant leaf disease identification approaches based on DCNN’s influence in the last 5 years. The usage of deep learning for plant leaf identification has been proved to be efficient and accurate. Shah et al. [117] investigated ResTS (Residual Teacher/Student)—a novel architecture for classification and visualization. ResTS first deconstructs input images using the ResTeacher classifier for latent representations and then reconstructs images using a decoder followed by a deconstruction of replicated images using ResStudent. Comparison with other state-of-the-art with the PlantVillage dataset containing 54,306 images of 14 different plants has shown promising results. Bedi et al. [118] presented a hybrid model comprised of convolutional autoencoder (CAE) and CNN for disease identification from peach plant leaves. The proposed model has used only a few parameters and provided a testing accuracy of 98.38% on the PlantVillage data set. On the other hand, Khanramaki et al. [119] presented an ensemble of CNNs and achieved an accuracy of 99.04% on 1774 citrus leaves.

Instead of developing a new CNN model from scratch, researchers are utilizing transfer learning, and the same is gaining tremendous popularity nowadays. In transfer learning, an existing CNN model for accomplishing a task is utilized for another study. In [120,121,122], researchers presented the concept of disease identification from plant leaf images using transfer learning. Sravan et al. [120] demonstrated the fine-tuning of hyperparameters of existing ResNet50 for disease classification and achieved a higher accuracy of 99.26% on the PlantVillage data set containing 20,639 images. In contrast, Jiang et al. [121] utilized VGG16 to recognize diseases from rice and wheat plants. Experimental evaluations and comparisons with other state-of-the-art presented an overall accuracy of 97.22% and 98.75% on rice and wheat plants.

On the other hand, Tahir et al. [122] investigated disease identification from the Apple plant using InceptionV3 and shown an overall accuracy of 97% on the PlantVillage data set. Shin et al. [123] depicted the comparative study of six different CNN models to identify powdery mildew disease on strawberry leaves. The optimal models for different parameters, videlicet, accuracy, speed, and hardware requirement have been suggested upon comparison. ResNet-50 provided the highest classification accuracy of 98.11%, AlexNet with the fastest processing time, and SqueezeNet-MOD2 with fewer memory requirements. Several implementations of DCNNs based on the concept of both novel architecture and transfer learning are presented in Table 6.

Instead of only identifying the presence or absence of diseases in plant leaves using deep learning, the severity of diseases is gaining popularity nowadays. Agarwal et al. [124] proposed a new Conv2D model for disease severity identification from the Cucumber plant. Comparison with not only other CNN models but also with machine learning models revealed improvements in disease identification.

## 4. Discussion and Conclusions

In this manuscript, the authors present a survey of the existing literature in identifying and classifying plant leaf diseases using Deep Convolutional Neural Networks (DCNN). The authors identified the closely related research articles for presenting the comparative analysis of different DCNN architectures. The survey focuses on the study of plant diseases, datasets used, size of the datasets, image pre-processing techniques, CNN architectures, CNN frameworks, performance metrics, and experimental results of different models applied to identify and classify plant leaf diseases.

For the classification of plant leaf diseases, the researchers applied traditional techniques of augmentation such as rotation [30,40], flipping, scaling, cropping, translation [18], and adding Gaussian noise [25]. They achieved satisfactory outputs using the above-stated techniques. Thus, the literature has observed the minimum use of deep learning augmentation techniques, such as Generative Adversarial Networks, Neural Style Transfer, etc.

Based on the literature, the authors conclude that DCNN is a better feature extractor than handcrafted feature engineering. DCNN being an automatic feature extractor saves the efforts of researchers. Furthermore, these models are robust under challenging conditions, such as images with a complex background, non-uniform size, improper orientation, and images captured in poor illumination, etc. However, there are several challenges and issues of these approached, videlicet, the requirement of a huge dataset, longer training time to yield the desired output. The current approaches reduced human intervention in predicting plant diseases, but the availability of a small dataset for training may lead to their poor prediction accuracy. Additionally, self-collection and labeling are tedious and time-consuming, requiring expertise in plant disease identification. Further, selecting optimum number layers and parameters is not defined according to the type and size of the dataset. As per the current research, the model’s architecture is designed based on the number and type of samples in a dataset. On the other hand, extracting the region of interest from the collected images is challenging, as the infected area may have low visibility due to occlusion while capturing the images.

The performance of DCNN models is directly proportional to the amount and accuracy of the labeled data used for training. If there is a low availability of data for training the model, then fine-tuned pre-trained models can give better results than the models trained from scratch.

Discussion of the common Deep Convolutional Neural Network (DCNN) architectures shows that selecting a suitable CNN or DCNN model depends on the type of dataset, size of the dataset, and experimental conditions. In the case of small network size and good accuracy, SqueezeNet is a good choice. As the number of layers increases, its efficiency is reduced. For deeper networks, ResNet is the better choice that uses skip connections. DenseNet architecture performs well where all the layers are connected directly with each other in a feed-forward manner. It reduces the vanishing-gradient problem and requires a few numbers of parameters.

Caffe is a high-speed and efficient framework that helps in building the Convolutional Neural Networks for image classification. Moreover, this tool allows applying Neural Networks without writing the code. Therefore, the Caffe framework is the most accessible framework to apply. The authors observed that around 33% of researchers from the literature review applied the Caffe framework to evaluate the performance of DCNNs.

The study of the existing literature reveals that researchers applied the CNN models on the images captured from the upper side of leaves, using cameras or drones. The existing research focuses on disease identification and classification but lacks in presenting information about the localization of disease regions. It highlights the advantages and challenges in applying computational technologies in agriculture and hence gives a direction towards developing feasible solutions for increasing crop productivity. This is completed by helping the researchers identify the suitable framework, architecture, model, optimization technique, and dataset for disease prediction. The early stage prediction of diseases helps apply preventive and disease-diagnosing measures.

There is also scope in the automatic estimation of the severity of plant diseases which may prove helpful to farmers in deciding what measures they need to take for the culmination of a disease.

## Figures and Tables

**Table 1:** Comparison of machine learning and deep learning models.

Points of Difference	Machine Learning Models	Deep Learning Models
Data Requirements	Require a small amount of data for training a model.	Require a large amount of data to train a model.
Hardware Dependency	Machine learning algorithms can work on low-end machines such as CPUs.	Deep learning models need high-end machines for execution, such as GPUs.
Feature Engineering	Machine learning models rely on hand-crafted feature extractors such as Histogram of Oriented Gradients (HOG), Scale-Invariant Feature Transform (SIFT), Speeded-Up Robust Features (SURF), Principle Component Analysis (PCA), etc. for extracting features from an image.	Do not require explicit identification of features from an image. Deep learning models perform automatic feature extraction without human intervention.
Interpretability	Machine learning algorithms such as decision trees give crisp rules to justify why and what the algorithm chooses. Thus, it is quite easy to interpret the reasoning behind these algorithms.	It is difficult to interpret the reasoning behind deep learning algorithms.
Training Time	It takes less time to train a model. The time ranges from a few minutes to a few hours. The training time is dependent on data size, hardware configuration, type of model, etc.	It takes more time to train a model. The time ranges from a few hours to a few weeks. The training time is dependent on data size, hardware configuration, type of model, number of layers in a model, etc.
Problem Solving Technique	Divides a problem into subproblems, solves each subproblem individually, and combines results obtained from each subproblem to solve the complete problem.	Efficient in providing a solution for the complete problem. Efficient in performing both feature extraction as well as classification.

**Table 2:** Comparison of pre-processing techniques.

Preprocessing Technique	Objective(s)	Methodology	Working Mechanism	Advantages	Disadvantages
Resizing	Effective utilization of storage space and reducing computation time.	Nearest-neighbor interpolation	Replaces the value of each input pixel with the translated value nearest to it.	Simple and fast.	Causes distortion, blurring, and edge halos.
		Bilinear interpolation	The average of four nearest pixel values is used to find the value of a new pixel.	No grey discontinuity defects and provides satisfactory results.	Produces blurring and edge halos. Time consuming and more complex than the nearest-neighbor interpolation.
		Bicubic interpolation	Considers the closest 4 × 4 neighborhood of known pixels, i.e., 16 nearest neighbors of a pixel.	Provides smoother images with less interpolation distortion.	It needs more time to generate the output due to complex calculations.
Augmentation	To increase the amount of relevant data in a dataset for training a model.	Traditional augmentation techniques	Generate new data from existing data by applying various transformation techniques such as rotation, flipping, scaling, cropping, translation, adding Gaussian noise, etc.	Simple to implement.	Disadvantages of geometric transformations include additional memory, transformation compute costs, and additional training time.
		Generative Adversarial Networks (GANs)	Comprise of a generator and a discriminator. Generator generates new examples, whereas discriminator distinguishes between generated and real.	Gives very impressive result by generating realistic visual content.	It fails to recover the texture of an image correctly. In the case of too small text or distortion in an original image, it generates a completely different image.
		Neural style transfer	Combines the content of one image with the style of another to form a new image.	Generating artistic artifacts with high quality.	
Normalization and Standardization	Used to find a new range of pixel values of an image.	Decimal scaling	Divides all pixel values with the largest value, i.e., 255 (8-bit RGB image).	Simplest transformation technique.	
		Min–Max normalization	The minimum pixel value is transformed to 0; the maximum value is transformed to 1. Other values are transformed into a decimal number between 0 and 1.	It provides a uniform scale for all pixels.	It is ineffective in handling outliers.
		Standardization or Z-score normalization	Standardization or Z-score normalization performs zero centering of data by subtracting the value of mean from each pixel and then dividing each dimension by its standard deviation.	It effectively handles outliers.	It does not produce normalized data with a uniform scale.
Annotation	Used for selecting objects in images and labeling the selected objects with their names.	Bounding box annotations	A rectangle superimposed over an image in which all key features of a particular object are expected to reside.	Easy to create, declared by simply specifying X and Y coordinates for the upper left and bottom right corners of the box.	Additional noise is also included in the bounded box. This method faces difficulty for occluded objects.
		Pixel-wise image annotations	Point-by-point object selection is completed through the edges of objects.	Easy to use for any task where sizable, discrete regions must be classified/recognized.	High computation cost in terms of time. More prone to human errors.
Outlier Rejection	Ignores invalid or irrelevant images from a dataset.	OrganNet	OrganNet is a CNN model, trained on the existing image datasets (ImageNet and PlantClef) as an automatic filter for data validation.	OrganNet is more efficient than the hand-design features set.	
Denoising	Noise removal from an image.	Gaussian filter	Blurs an image and removes noise using a Gaussian function.	Conceptually simple, reduces noise and edge blurring.	It takes time, images are blurred as image details and edges are degraded.
		Mean filter	It is a linear filter that replaces the center value in the window with the mean or average of all values of the pixel in the window.	Simple, easy to implement for smoothing of images.	Over-smooth images with high noise.
		Median filter	It is a non-linear filter that replaces the center value in the window with the median of all values of the pixel in the window.	Reduces noise. Better than mean filter in preserving sharp edges.	Relatively costly and complex to compute.
		Wiener filter	It minimizes the overall mean square error in the process of inverse filtering and noise smoothing.	It is optimal in terms of mean square error. Removes the additive noise and inverts the blurring simultaneously.	Slow to apply; blurs sharp edges.
		Bilateral smoothing filter	It replaces the intensity of each pixel with a weighted average of intensity values from nearby pixels.	Preserves edges. Reduces noise. Performs smoothing.	Less efficient.

**Table 3:** Comparison of popular CNN architectures.

Architecture	Layers	Parameters	Highlights	Reference
AlexNet	8 (5 Convolution + 3 Fully Connected)	60 million	AlexNet is similar to LeNet-5, but it is deeper, contains more filters in each layer, and uses stacked convolutional layers. Winner of ILSVRC-2012.	[74]
VGGNet	16–19 (13–16 convolution + 3 FC)	134 million	The depth of a model is increased by using small convolutional filters of dimensions 3 × 3 in all layers to improve its accuracy. First runner-up in ILSVRC-2014 challenge.	[31]
GoogLeNet	22 Convolution layers, 9 Inception modules	4 million	A deeper and wider architecture with different receptive field sizes and several very small convolutions. Winner of ILSVRC-2014.	[78]
Inception v3	42 Convolution layers, 10 Inception modules	22 million	Improves the performance of a network. It provides faster training with the use of Batch Normalization. Inception building blocks are used in an efficient way for going deeper.	[29]
Inception v4	75 Convolution layers	41 million	Inception-v4 is considerably slower in practice due to many layers.	[108]
ResNet	50 in ResNet-50, 101 in ResNet-101, 152 in ResNet-152	25.6 million in ResNet-50, 44.5 million in ResNet-101, 60.2 million in ResNet-152.	A novel architecture with ‘skip connections’ and heavy batch normalization. Winner of ILSVRC 2015.	[32]
ResNeXt-50	49 Convolution layers and 1 Fully Connected layer	25 million	Use ResNeXt blocks based on the strategy of ‘split–transform–merge’. Despite creating filters for a full channel depth of input, the input is split into groups. Each group represents a channel.	[81]
DenseNet-121	117 Convolution layers, 3 Transition layers and 1 Classification layer	27.2 million	All layers are connected directly with each other in a feed-forward manner. It reduces the vanishing-gradient problem and requires few parameters.	[33]
SqueezeNet	Squeeze layer and Expand layers	50 times fewer parameters than AlexNet.	SqueezeNet is a lightweight model of size 2.9 MB. It is approximately 80 times smaller than AlexNet. Achieves the same level of accuracy as AlexNet. Reduces the number of parameters by using a smaller number of filters.	[83]
LeNet-5	7 (5 Convolution + 2 FC)	60 thousand	Fast to deploy and efficient in solving small-scale image recognition problems.	[73]

**Table 4:** Advantages and disadvantages of various optimization techniques.

Name of Optimizer	Advantages	Disadvantages
BGD	Easy to compute, implement and understand.	It requires large memory for calculating gradients on the whole dataset. It takes more time to converge to minima as weights are changed after calculating the gradient on the whole dataset. May trap to local minima.
SGD	Easy to implement. Efficient in dealing with large-scale datasets. It converges faster than batch gradient descent by frequently performing updates. It requires less memory as there is no need to store values of loss functions.	SGD requires a large number of hyper-parameters and iterations. Therefore, it is sensitive to feature scaling. It may shoot even after achieving global minima.
AdaGrad	Learning rate changes for each training parameter. Not required to tune the learning rate manually. It is suitable for dealing with sparse data.	The need to calculate the second-order derivative makes it expensive in terms of computation. The learning rate is constantly decreasing, which results in slow training.
RMSProp	A robust optimizer has pseudo curvature information. It can deal with stochastic objectives very nicely, making it applicable to min-batch learning.	The learning rate is still handcrafted.
Adam	Adam is very fast and converges rapidly. It resolves the vanishing learning rate problem encountered in AdaGrad.	Costly computationally.

**Table 5:** Comparative analysis of CNN frameworks.

Framework	Compatible Operating System	Programming Language Used for Development	Interface	Open Source	OpenMP Support	OpenCL Support	CUDA Support
TensorFlow	Linux, macOS, Windows, Android	C++, Python, CUDA	Python, Java, Go, JavaScript, R, Swift, Julia	Yes	No	Build TensorFlow with Single Source OpenCL	Yes
Theano	Cross-platform	Python	Python	Yes	Yes	Under development	Yes
Keras	Linux, macOS, Windows	Python	R, Python	Yes	Yes	TensorFlow as backend	Yes
Caffe	Linux, macOS, Windows	C++	C++, MATLAB, Python	Yes	Yes	Under development	Yes
Torch	Linux, macOS, Windows, Android, iOS	C, Lua	Lua, LuaJIT, C, C++/OpenCL	Yes	Yes	Third-party implementations	Yes
deeplearning4j	Linux, macOS, Windows, Android	Java, C++	Java, Scala, Python, Clojure, Kotlin	Yes	Yes	No	Yes
DL Matlab Toolbox	Linux, macOS, Windows	MATLAB, Java, C, C++	MATLAB	No	No	No	Via GPU Coder

**Table 6:** Comparative analysis of DCNN in identification of plant leaf diseases.

Plant	Disease	Architecture	Datasets	Results
Banana	Black sigatoka and Black speckle	LeNet [21]	PlantVillage: 3700 images	Accuracy: 99%
Apple	Black rot on Apple leaves	VGG16, VGG19, Inception-v3 and ResNet50 [18]	PlantVillage: 2086 images	VGG16: 90.4%, VGG19: 90.0%, Inception-v3: 83.0%, ResNet50: 80.0%
14 different crop species	26 different diseases	AlexNet, GoogLeNet [23]	PlantVillage: 54,306 images	AlexNet: Accuracy: 99.28% GoogLeNet: Accuracy: 99.35%
6 different fruit plant species	13 different diseases	Modified CaffeNet [25]	Authors created database containing 4483 images downloaded from the internet	Accuracy: 96.3%
Tomato	9 different diseases in tomato	AlexNet, GoogLeNet [35]	PlantVillage: 14,828 Images	GoogleNet: Accuracy: 99.18% AlexNet: Accuracy: 98.66%
Cucumber	Melon Yellow Spot Virus (MYSV), Zucchini Yellow Mosaic Virus (ZYMV)	Author-defined CNN [36]	800 images of cucumber leaves captured by Saitama Prefectural Agriculture and Forestry Research Center, Japan	Average accuracy: 94.9%, MYSV Sensitivity: 96.3%, ZYMV Sensitivity: 89.5%,
Rice	10 different diseases	Author-defined CNN [19]	The author created a database of 500 images captured from experimental rice fields of Heilongjiang Academy of Land Reclamation Sciences, China	Accuracy: 95.48%
Tomato	9 different types of diseases and pests	VGG-16, ResNet-50, ResNet-101, ResNet-152, ResNetXt-50, [82]	The author created a dataset of 5000 images captured through a camera from tomato farms located in Korea	VGG-16: 83.06%, ResNet-50: 75.37%, ResNet-101: 59.0%, ResNet-152: 66.83%, ResNetXt-50: 71.1%
25 different Plant’s species	19 different plant diseases	AlexNet, AlexNetOWTBn, GoogLeNet, Overfeat and VGGNet [24]	PlantVillage: 87,848 images of different plants (Both laboratory and field conditions)	AlexNet: 99.06%, AlexNetOWTBn: 99.49%, GoogLeNet: 92.27%, Overfeat: 98.96%, VGGNet: 99.53%
Apple	Mosaic, Rust, Brown spot, and Alternaria leaf spot	Authors-defined CNN architecture based on AlexNet [39]	Dataset of 13,689 synthetic images	Proposed Model: 97.62%, AlexNet: 91.19%, GoogLeNet: 95.69%, ResNet-20: 92.76%, VGGNet-16: 96.32%
Olive	Olive Quick Decline Syndrome (OQDS)	Authors-defined LeNet [28]	PlantVillage	Accuracy of 99%
Tomato	9 different types of diseases of tomato plant	AlexNet and SqueezeNet [40]	PlantVillage	AlexNet: 95.65%, SqueezeNet: 94.3%
Wheat	6 different diseases of wheat	VGG-CNN-S, VGG-CNN-VD16, VGG-FCN-S and VGG-FCN-VD16 [38]	WDD2017: 9230 wheat crop images	VGG-FCN-VD16: 97.95%, VGG-FCN-S: 95.12%, VGG-CNN-VD16: 93.27%, VGG-CNN-S: 73.00%
Cucumber	Anthracnose, Downy mildew, powdery mildew and Target leaf spots	Architecture similar to LeNet-5 [37]	1184 images: PlantVillage, forestry and captured through digital camera	Proposed model: 93.4%, SVM: 81.9%, RF: 84.8%, AlexNet: 94.0%
Radish	Fusarium wilt	VGG-A [26]	139 Images captured by a commercial UAV equipped with an RGB camera	Accuracy: 93.3%
14 different plant species	79 different diseases	GoogLeNet [65]	1567 images captured using smartphones, compact cameras, DSLR cameras	Average accuracy: 94%
Potato	Black Scurf disease, Silver Scurf, Common Scab and Black Dot disease	VGG [22]	A total of 2465 patches of diseased potatoes	Accuracy: 96.00%
Tomato	Early Blight, Late Blight, Yellow Leaf Curl Virus, Spider Mite Damage and Bacterial Spot	VGG-19, Xception, Inception-v3, ResNet-50 [59]	PlantVillage: 3750 images	ResNet-50: 99.7%, Xception: 98.6%, Inception-v3: 98.4%, VGG-19: 98.2%
Wheat	Septoria, Tan Spot and Rust	ResNet50 [68]	Author-defined dataset of 8178 images	Accuracy: 96.00%
Cassava	3 diseases: Brown leaf spot, Brown streak, and cassava mosaic 2	Inception-v3 [104]	Author-defined dataset. Originally: 2756 images. Leaflet: 15,000 images	Accuracy: 93.00%
14 different plant species	Not mentioned	VGG 16, Inception V4, ResNet50, ResNet101, ResNet152 and DenseNet121 [20]	PlantVillage	VGG16: 82%, Inception V4: 98%, ResNet50: 99.6%, ResNet101: 99.6%, ResNet152: 99.7% and DenseNet121: 99.75%
Maize	8 different diseases	GoogLeNet and Cifar10	500 images were collected from different sources: Plant Village and Google websites	GoogLeNet: 98.9% Cifar10: 98.8%
Wheat	6 different diseases	Author-defined architecture named M-bCNN (Matrix-based CNN) [85]	16,652 images collected from Shandong Province, China	Accuracy: 90.1%
Maize	Northern Leaf Blight	Five CNNs were trained on the augmented data set with variations in the architecture and hyperparameters of the networks [64]	1796 images of maize leaves grown on the Musgrave Research Farm in Aurora, NY	Accuracy: 96.7%,
Apple	6 different diseases	AlexNet [106]	2539 images of three species of apple trees from orchards located in the southern part of Brazil	Accuracy: 97.3%,
Radish	Fusarium wilt of radish	GoogLeNet [102]	The images were captured in Korea, including Jungsun, Gangwon, and Hongchun, using two commercial UAVs	Accuracy: 90%
Tomato	8 different diseases	AlexNet, GoogLeNet, and ResNet [60]	PlantVillage: 5550 images	ResNet: 97.28%
Rice	Rice Blast Disease	Two CNN models similar to Lenet5 [86]	5808 images are obtained from the Institute of Plant Protection, Jiangsu Academy of Agricultural Sciences, Nanjing, China	First CNN: 95.37% Second CNN: 95.83%
Banana	Five major diseases along with a pest class	ResNet50, InceptionV2, and MobileNetV1 [103]	Dataset comprises about 18,000 field images of bananas from Bioversity International, Africa, and Tamil Nadu Agricultural University, India	Accuracy between 70–90%
Apple, Banana	Apple scab, apple rot, banana sigotka, banana cordial leaf spot, banana diamond leaf spot, and Deightoniella leaf and fruit spot	VGG-16 [27]	6309 sample images of apple and banana fruits PlantVillage and CASC-IFW datasets	Accuracy: 98.6%
Grapevine	Esca disease	LeNet-5 [77]	The dataset consists of 70,560 learning patches by the UAV system with an RGB sensor	The best results were obtained with the combination of ExR, ExG and ExGR vegetation indices using (16 × 16) patch size reaching 95.80%
Maize	The northern corn leaf blight, common rust and gray leaf spot	Author-defined CNN [61]	PlantVillage	Accuracy: 92.85%
Tea	7 Diseases: Red leaf spot, Algal leaf spot, Bird’s eye spot, Gray blight, White spot, Anthracnose, Brown blight	Author-defined CNN model named LeafNet (Improvement over AlexNet) [84]	A total of 3810 tea leaf images captured using a Canon PowerShot G12 camera in the natural environments of Chibi and Yichang within the Hubei province of China.	Accuracy: 90.16%
